# Trend of metabolic risk factors among the population aged 25–64 years for non-communicable diseases over time in Vietnam: A time series analysis using national STEPs survey data

**DOI:** 10.3389/fpubh.2022.1045202

**Published:** 2022-11-30

**Authors:** Lan Thi Hoang Vu, Quyen Thi Tu Bui, Long Quynh Khuong, Bao Quoc Tran, Truong Duc Lai, Minh Van Hoang

**Affiliations:** ^1^Faculty of Fundamental Science, Hanoi University of Public Health, Hanoi, Vietnam; ^2^Faculty of Science, University of Hasselt, Hasselt, Belgium; ^3^General Department of Preventive Medicine, Ministry of Health (Vietnam), Hanoi, Vietnam; ^4^World Health Organization Country Office for Viet Nam, Hanoi, Vietnam; ^5^Hanoi University of Public Health, Hanoi, Vietnam

**Keywords:** NCD, metabolic risk factor, time series analyses, trend, STEPs

## Abstract

**Introduction:**

The study aims to examine the trends of 4 metabolic NCDs risk factors including raised blood pressure, increased blood glucose, elevated blood lipids and overweight/obesity over the last 10 years in Vietnam as well as examine these trends among different sub-population by geographical area, gender, and age groups.

**Methods:**

The study combined the national representative data from three rounds of STEPs survey in Vietnam conducted in 2010, 2015, and 2020 on people aged 25–64 years. The overall prevalence of each metabolic factor together with 95% CI for each time point as well as the stratified prevalence by rural/urban, male/female, and 4 separated age groups were calculated and considered the sampling weight. Cochran–Armitage test for trend was used to test for the differences in the prevalence over time.

**Results:**

The prevalence of hypertension, overweight/obesity, hyperglycemia, and hyperlipidemia among the population aged 25–64 years old was 28.3, 20.57, 6.96, and 15.63%, respectively in the year 2020. All NCD metabolic risk factors examined in this analysis show significantly increasing trends over time. For most age groups, the increasing burden of NCD metabolic risk factors was more significant during the period 2015–2020 compared to the period 2010–2015. Male population and population aged 55–64 experienced the most dramatic changes in the burden of all NCD metabolic risk factors.

**Conclusion:**

To reverse the increasing trend of NCD metabolic factors in Vietnam, intervention, and policy need to apply a comprehensive life course approach.

## Introduction

Non-communicable diseases (NCDs) are one of the leading causes of death worldwide in both developing and developed countries. In addition, its incidence and mortality have an increasing trend in most countries around the world; it is responsible for 73% of the total deaths in 2018 (1). During 2010–2020, the mortality burden due to NCDs increased by 15% (equivalent to 44 million deaths). More significantly, deaths from NCDs in low-income countries will be eight times higher than those in developed countries in 2030 (2).

Vietnam achieved significant changes in the economic situation and became a middle-income country in 2008. Along with economic growth, Vietnam is also experiencing fast and wide urbanization and a population aging process. The rapid epidemiological and demographic transition in the last 20 years has resulted in a significant increase in the burden of NCDs in Vietnam. It is estimated that in 2016, the whole country had 549,000 deaths of all kinds, of which 77% were deaths due to NCDs, mainly cardiovascular diseases (31%), cancer (19%), diabetes (4%), and chronic obstructive pulmonary disease (6%) (1, 3).

With the high level of risk factors driven by the increasingly global economy and fast population aging, NCDs are expected to worsen in the future. For that reason, NCDs prevention and control has been one of Vietnam's health priorities (4). As many other countries, Vietnam has applied the STEPs survey introduced by WHO (5) as an effective information system to monitor trends of NCDs and their risk factors to provide evidence for developing policies and related interventions.

The STEPs survey is a comprehensive approach to measuring established core risk factors responsible for NCDs at the population level. The survey consisted of three steps: (1) STEP 1 was for collecting demographic information/behavioral risk factors in an interviewer-administered survey; (2) STEP 2 was for collecting physical measurements such as height/weight/blood pressure, and (3) STEP 3 was for obtaining blood samples to test for glucose/cholesterol and urine samples.

Vietnam completed three rounds of the STEP survey in the years 2010, 2015, and 2020. All previous studies using STEPs 2010 and 2015 data was cross-sectional study, thus, was not able to present and test for the significant changes of the patterns of NCD metabolic factors over time (6–8). This study attempts to combine the data from the three-round surveys to explore the trend of 4 metabolic NCDs risk factors, including raised blood pressure, overweight/obesity; increased blood glucose; and elevated blood lipids over the last 10 years in Vietnam as well as examine these trends among different sub-population by geographical area, gender, and age groups.

## Methods

### Data sources

The 2010 survey applied a three-stage sampling method. First, eight provinces were randomly selected, representing eight ecological regions of Vietnam. Within each province, 20 clusters were randomly selected (commune as sampling unit). Within each commune, study subjects were randomly selected. Stratified sampling by gender, age groups, and rural/urban was applied. Of the 22,940 eligible subjects selected for participation, 14,706 (64.1%) participated in this survey. The sampling method and the characteristics of the 2010 survey was presented in a previous study (6).

The 2015 step survey used a two-stage random systematic sampling method. A household's primary sampling unit (PSU) was identified in the first stage. The sample frame was 15% of the general population of Vietnam and represented all 63 provinces and cities. The original samples were stratified by gender and age group (18–29, 30–49, and 50–69 years). Of the 3,856 eligible subjects selected for participation, 2,816 participants had completed all stages of STEPs survey (response rate 73.0%). Details of the 2015 survey have been reported elsewhere (7).

The 2020 survey used the same sampling approach as the 2015 survey. The sample was based on the National Sampling Master Frame developed by the General Statistics Office (GSO). The PSU was Enumeration Areas (EAs). Of the 5,000 eligible subjects selected for participation, 3,712 subjects had completed all three stages of STEPs survey (response rate of 74.2%).

### Study population

The study population for the STEPs survey in 2015 and 2020 was Vietnamese persons aged 18–69 years. The survey population in the year 2010, however, included only persons aged 25–64 years. To keep the survey comparable over three-time points, we selected data for the population aged 25–64 years in all three surveys. Thus, the selection criteria for this analysis were Vietnamese persons aged 25–64 years who were residing in Vietnam at the time of the survey. Exclusion criteria included (1) those who were not residing permanently in Vietnam, (2) in-patients who were treated at health facilities, and (3) people with impaired mental health. The survey was approved by all participating institutions. All survey participants were provided verbal and/or written informed consent.

### Key measurement

The NCD's metabolic risk factors examined in this analysis included both physical and biochemical measurements. Physical measurements were (1) body mass index (BMI) calculated as weight (kg)/height^2^ (m); (2) Blood pressure (BP) measured by cuff at the midpoint of the right upper arm by trained staff using a validated digital BP monitor with participants seated and rested for at least 15 min (two BP readings were taken 3 min apart, and third reading was taken if there was a difference between the two readings of more than 25 mmHg for systolic BP or more than 15 mmHg for diastolic BP. If a third measurement was taken, the mean of the two closest measures was used; otherwise, the mean of the two measures was used). Biochemical measurements were: (1) total cholesterol (TC) and (2) blood glucose (BG) from whole capillary blood after overnight fasting.

Overweight/obesity was defined as study subjects with BMI ≥ 25.

Raised blood pressure: defined study subjects with SBP ≥140 mmHg, and/or DBP ≥90 mmHg, and/or currently on medicine for raised blood pressure.

Increased blood glucose: BG from whole capillary blood after overnight fasting ≥7 mmol/l and/or currently on medicine for diabetes.

Elevated blood lipids: TC from whole capillary blood after overnight fasting ≥6.2 mmol/l and/or currently on medicine for elevated blood lipids.

### Statistical approach

Weights were calculated for STEPS 1, STEPS 2, and 3 separately. Base weight was first calculated based on the inverse of the probability of selection, and then a non-response adjustment was made for non-response at household and individual levels. Census data in relevant years was used to estimate the population aged 25–64 years. The population adjustment was made for 16 subgroups obtained from males-females; urban-rural, and four age groups: 25–34; 35–44; 45–54; 55–64 years.

The survey data (SVY) procedure in STATA 17 was used to estimate the overall prevalence of raised blood pressure, overweight/obesity, increased blood glucose, and elevated blood lipids. Suitable weights were selected for different outcomes. For raised blood pressure and overweight/obesity, STEP 2 weights were used for all the estimation. For increased blood glucose and elevated blood lipids. STEP 3 weights were used for all the calculation.

For all metabolic risk factors, the study estimated the weighted prevalence, 95% CI for year as well as the stratified prevalence's by rural/urban, male/female, and 4 separated age groups. The differences in burden of metabolic factors between male/female, urban/rural and across 4 age groups were tested by Chi-square (*p* < 0.05 indicated significant differences). Cochran–Armitage test for trend was used to test for the differences in the prevalence over time. The Cochran- Armitage was performed by using nptrend command in STATA. This procedure has an option to compute exact *p*-values based on Monte Carlo permutations (*p* < 0.05 indicated significant trend over time).

The logistic regression for survey data (i.e., svy: logit in STATA) was used to examine whether the trends of some groups varied over time. For instance, a logistics model for dependent variable raised blood pressure (1: yes, 0: no) with three independent variables: year, gender and product term of year and gender was used to test whether the trend of raised blood pressure among female population were different from that among male population. If the *p*-value of the product term (i.e., year^*^ gender) was <0.05, these two trends were statistically different.

## Results

### Changes in the prevalence of raised blood pressure among the population aged 25–64 years over time

In 2020, the prevalence of raised blood pressure was 28.33% (95% CI: 26.34%; 30.32%).

Table 1 presents the prevalence of raised blood pressure over time and the differences between 2015/2010 and 2020/2015. The prevalence of raised blood pressure for all populations and for all sub-groups increased significantly over time (Cochran–Armitage test for trend, *p* < 0.001). The increasing trend was more significant between the years 2020 and 2015 than between the years 2015 and year 2010. Specifically, the absolute difference in the prevalence of raised blood pressure between 2010 and 2015 was only 4.94%, while this was 7.55% between 2015 and 2020.

**Table 1:** Absolute changes in the prevalence of raised blood pressure among the population aged 25–64 years over time.

**Characteristics**	**Year 2010,** **Proportion (%)** **[95%CI]**	**Year 2015,** **Proportion (%)** **[95%CI]**	**Year 2020,** **Proportion (%)** **[95%CI]**	**Absolute differences** **(%, p2–p1)**		***p*-value from Cochran–Armitage test for trend**
				**Between** **2010 – 2015**	**Between** **2015 – 2020**	
All	15.85 [15.04; 16.65]	20.78 [18.95; 22.60]	28.33 [26.34; 30.32]	4.93	7.55	< 0.001
**Urban/rural**
Urban	14.87 [13.36; 6.38]	20.33 [17.59; 23.06]	30.6 [27.12; 34.09]	5.46	10.27	< 0.001
Rural	16.24 [15.25; 17.22]	21.01 [18.62; 23.41]	27.04 [24.73; 29.35]	4.77	6.03	< 0.001
**Gender**
Male	19.81 [18.68; 20.94]	25.09 [22.01; 28.17]	36.75 [33.65; 39.84]	5.28	11.66	< 0.001
Female	12.05 [11.22; 12.89]	16.67 [14.48; 18.86]	20.06 [17.76; 22.36]	4.62	3.39	< 0.001
**Age groups**
Age 25–34	5.86 [4.93; 6.80]	5.73 [3.49; 7.97]	10.69 [7.67; 13.70]	−0.13	4.96	< 0.001
Age 35–44	13.25 [11.99; 14.50]	15.15 [12.22; 18.08]	23.89 [18.90; 28.89]	1.90	8.74	< 0.001
Age 45–54	23.24 [21.73; 24.74]	31.76 [27.76; 35.76]	33.08 [28.97; 37.19]	8.52	1.32	< 0.001
Age 55–64	35.86 [33.94; 37.79]	42.36 [37.61; 47.11]	55.26 [51.62; 58.90]	6.50	12.90	< 0.001

For the rural population, the prevalence of raised blood pressure increased from 16.24% in 2010 to 21.01% in 2015 and 27.0% in 2020. For the urban population, the prevalence was 14.87%, 20.33%, and 30.6% in 2010, 2015, and 2020, respectively. Both urban and rural populations showed a significant increase in the prevalence of raised blood pressure over time. However, figures for urban demonstrates significantly higher increasing trend compared to that of rural (*p*-value = 0.027).

Data among the male population indicates much more dramatic changes over time compared to the female population (*p*-value for difference in trend between male and female=0.014). Among 4 age groups, the oldest group (aged 55–64 years) experiences the sharpest increase in the prevalence of raised blood pressure over time. For the age group 25–34 years and the age group 35–44 years, the differences between the years 2015 and 2020 were significantly higher than that between the year 2010–2015 (*p* = 0.018). However, for the age group 45–54 years, the difference between the years 2010-2015 was more noticeable compared to that between the years 2015–2020 (8.53 vs. 1.32%).

Four age groups showed significant differences in trend of raised blood pressure over time (*p*-value < 0.001).

**Figure 1:**
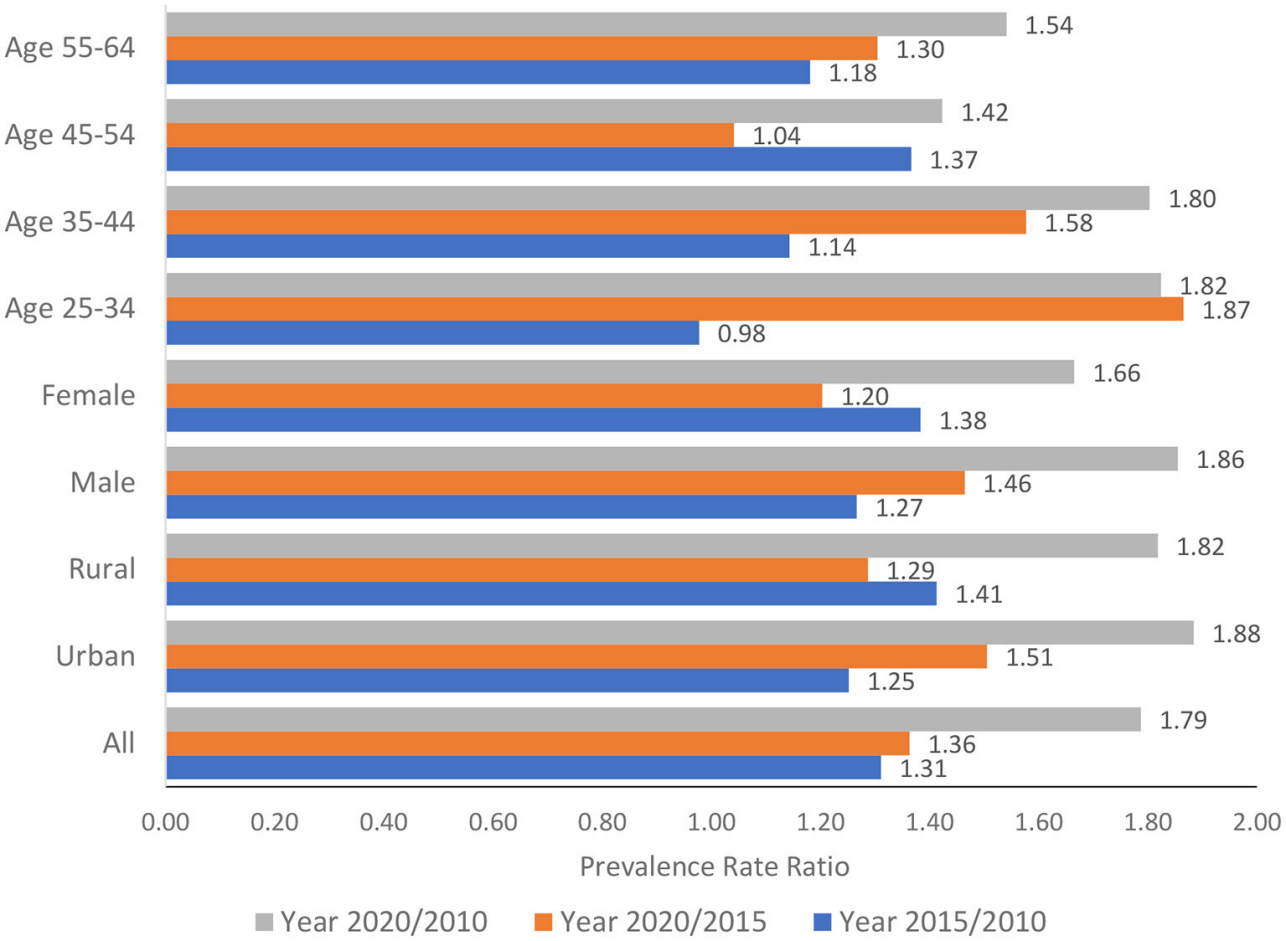
Relative changes in the prevalence of raised blood pressure among the population aged 25–64 years over time.

Figure 1 presents the relative changes in the prevalence of raised BP among all populations aged 25–64 years over time and among subgroups by age group, gender, and urban/rural The relative changes were measured by the prevalence rate ratios (PRR) between 2015 and 2010, 2020 and 2015, and 2020 and 2010. If the ratio was >1, the burden of raised BP would increase over time. It can be observed that during the last 10 years (i.e., from 2010 to 2020), the burden of raised BP doubled among the population aged 25–64 years in the urban population.

### Changes in the prevalence of overweight/obesity among the population aged 25–64 years over time

Table 2 examines the changes in the prevalence of overweight/obesity over the last 10 years. The overall prevalence of overweight/obesity for all populations and for all sub-groups increased significantly over time (Cochran–Armitage test for trend, *p* < 0.001). In the year 2010, the prevalence of the population aged 25–64 years having BMI ≥25 (i.e., overweight/obesity) was 10.48%; this figure went almost doubled in the year 2020 (20.57%). The trends were not statistically significant between rural and urban (*p*-value = 0.17) and between males and females (*p*-value = 0.57). Among 4 age groups, the youngest group (aged 25–34 years) experienced the most dramatic changes in the burden of overweight/obesity. Trends of overweight/obesity among 4 age groups were statistically different (*p*-value = 0.015).

**Table 2:** Changes in the prevalence of overweight/obesity over time.

**Characteristics**	**Year 2010,** **Proportion (%)** **[95%CI]**	**Year 2015,** **Proportion (%)** **[95%CI]**	**Year 2020,** **Proportion (%)** **[95%CI]**	**Absolute differences** **(%, p2–p1)**		***p*-value from Cochran–Armitage test for trend**
				**Between** **2010 – 2015**	**Between** **2015 – 2020**	
All	10.48 [9.68; 11.29]	17.63 [15.67; 19.59]	20.57 [18.45; 22.68]	7.15	2.94	< 0.001
**Urban/rural**
Urban	15.9 [13.94; 17.86]	23.08 [19.76; 26.40]	25.86 [22.68; 29.04]	7.18	2.78	< 0.001
Rural	8.32 [7.41; 9.22]	14.75 [12.37; 17.14]	17.58 [14.72; 20.43]	6.43	2.83	< 0.001
**Gender**
Male	10.84 [9.78; 11.91]	17.01 [14.16; 19.86]	20.19 [17.51; 22.86]	6.17	3.18	< 0.001
Female	10.14 [9.18; 11.09]	18.24 [15.81; 20.66]	20.94 [17.59; 24.29]	8.10	2.70	< 0.001
**Age groups (in years)**
25–34	7.85 [6.64; 9.05]	13.56 [9.91; 17.22]	20.06 [15.18; 24.95]	5.71	6.50	< 0.001
35–44	10.01 [8.78; 11.24]	17.43 [14.23; 20.63]	18.89 [15.85; 21.93]	7.42	1.46	< 0.001
45–54	13.15 [11.88; 14.41]	21.02 [17.63; 24.42]	21.56 [18.08; 25.04]	7.87	0.54	< 0.001
55–64	13.81 [12.17; 15.46]	20.62 [16.60; 24.65]	22.51 [19.28; 25.74]	6.81	1.89	< 0.001

Overall, the period from 2010 to 2015 had a higher increase rate of overweight/obesity compared to the period from 2015 to 2020 (7.15 vs. 2.94%). This pattern was observed for almost all sub-population groups, except for the population aged 25–34 years, as the difference between the years 2010–2015 was 5.71%, but the difference between the years 2015–2020 was 6.5%.

**Figure 2:**
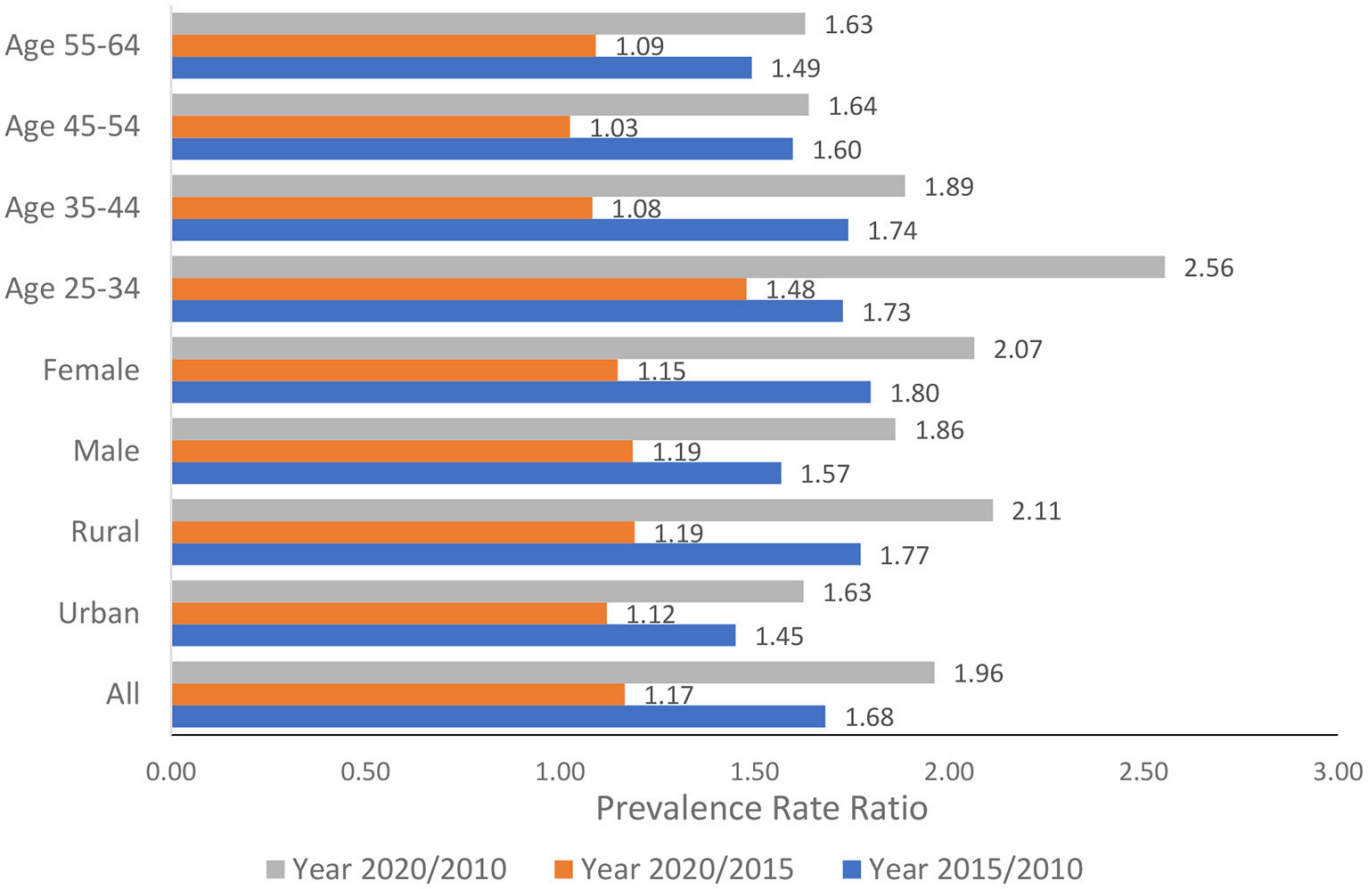
Relative changes in the prevalence of overweight/obesity among the population aged 25–64 years over time.

Figure 2 presents the relative changes in the burden of overweight/obesity among all populations aged 25–64 years over time and among subgroups by age groups, gender, and geographical areas. All the PRR for the years 2020/2010 was >1.5. Three groups, females aged 25–64 years, rural population, and population aged 25–34 years, had the PRR ≥ 2, showing an outbreak in the burden of overweight/obesity.

### Changes in the prevalence of increased blood glucose among the population aged 25–64 years over time

The changes over time in the prevalence of increased blood glucose (BG) among the population aged 25–64 years are presented in Table 3. This prevalence increased from 1.19% (95% CI: 0.89%; 1.48%) to 3.87% (95% CI: 3.05%; 4.70%) in 2015 and to 6.96% (95% CI: 5.89%; 8.03%) in 2020. The population aged 55–64 years experienced the sharpest increase in the burden of increased BG during the period 2015–2020 (i.e., from 7.59 to 15.07%). The Cochran–Armitage test shows that the increasing trends of increased BG for all populations and all sub-groups were statistically significant (*p* < 0.001).

**Table 3:** Changes in the prevalence of increased blood glucose over time.

**Characteristics**	**Year 2010,** **Proportion (%)** **[95%CI]**	**Year 2015,** **Proportion (%)** **[95%CI]**	**Year 2020,** **Proportion (%)** **[95%CI]**	**Absolute differences** **(%, p2–p1)**		***p*-value from Cochran–Armitage test for trend**
				**Between** **2010 – 2015**	**Between** **2015 – 2020**	
All	1.19 [0.89; 1.48]	3.87 [3.05; 4.70]	6.96 [5.89; 8.03]	2.68	3.09	< 0.001
**Urban/rural**
Urban	1.75 [0.86; 2.64]	5.56 [3.90; 7.21]	9.03 [7.21; 10.86]	3.81	3.47	< 0.001
Rural	0.96 [0.76; 1.17]	2.99 [2.09; 3.89]	5.85 [4.56; 7.14]	2.03	2.86	< 0.001
**Gender**
Male	1.04 [0.80; 1.29]	4.16 [2.94; 5.39]	7.85 [6.22; 9.48]	3.12	3.69	< 0.001
Female	1.32 [0.88; 1.77]	3.6 [2.60; 4.60]	6.11 [4.76; 7.46]	2.28	2.51	< 0.001
**Age groups (in years)**
25–34	0.71 [0.02; 1.40]	1.05 [0.29; 1.81]	2.29 [0.55; 4.03]	0.34	1.24	< 0.001
35–44	0.78 [0.47; 1.09]	3.67 [2.15; 5.19]	6.36 [3.86; 8.85]	2.89	2.69	< 0.001
45–54	1.59 [1.20; 1.98]	5.1 [3.38; 6.83]	6.77 [4.73; 8.80]	3.51	1.67	< 0.001
55–64	2.74 [2.13; 3.35]	7.59 [5.03; 10.16]	15.07 [12.43; 17.70]	4.85	7.48	< 0.001

The burden of increased BG among the urban population was steadily higher than that among the rural population over time, and the gap between urban/rural disease burdens also became bigger over time. The male and female populations all experienced a higher burden of increased BG over time, but the differences between males/females did not change.

**Figure 3:**
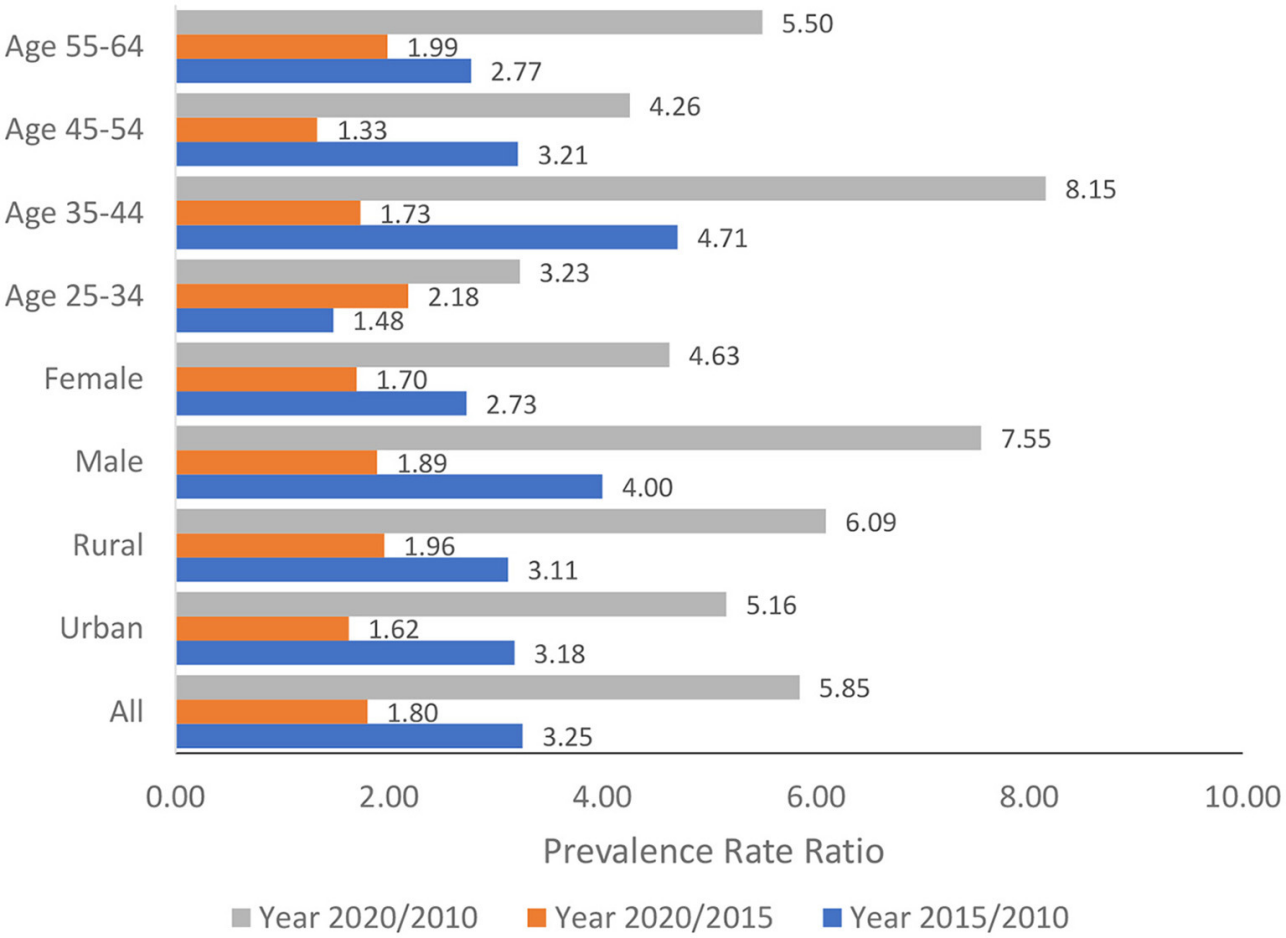
Relative changes in the prevalence of increased blood glucose over time.

The relative changes in the burden of increased BG over time among the population aged 25–64 years and for all the sub-groups analyses measured by the prevalence rate ratio were presented in Figure 3. Overall, the Vietnam population aged 25–64 years experienced a sharp increase in the burden of increased BG as the prevalence of increased BG in the year 2020 was 5.85 times higher than that in the year 2015. Sub-group analysis showed that among the male population and population aged 35–44 years, the prevalence of increased BG in the year 2020 was 7.55 times and 8.15 times higher than that in the year 2010.

In term of difference in trends over time, there was no statistically significant difference in trends between rural and ruban (*p* = 0.43), male and female (*p* = 0.17) and among four age groups (*p* = 0.44).

### Changes in the prevalence of elevated blood lipids among the population aged 25–64 years over time

Table 4 presents the prevalence of elevated blood lipids over time and the absolute differences between the years 2015/2010 and the year 2020/2015. The prevalence of elevated blood lipids among the population aged 25–64 years was 4.46% (95% CI: 4.01%; 4.90%). This figure went up to 10.31% (95%CI: 8.69%; 11.94%) in year 2015 and to 15.63% (95%CI: 13.65%; 17.61%) in year 2020. The Cochran–Armitage test shows that the increasing trends of elevated blood lipids for all populations and all sub-groups were statistically significant.

**Table 4:** Absolute changes in the prevalence of elevated blood lipids over time.

**Characteristics**	**Year 2010,** **Proportion (%)** **[95%CI]**	**Year 2015,** **Proportion (%)** **[95%CI]**	**Year 2020,** **Proportion (%)** **[95%CI]**	**Absolute differences** **(%, p2–p1)**		***p*-value from Cochran–Armitage test for trend**
				**Between** **2010 – 2015**	**Between** **2015 – 2020**	
All	4.46 [4.01; 4.90]	10.31 [8.69; 11.94]	15.63 [13.65; 17.61]	5.85	5.32	< 0.001
**Urban/rural**
Urban	6.84 [5.75; 7.93]	10.91 [8.55; 13.27]	18.92 [15.99; 21.85]	4.07	8.01	< 0.001
Rural	3.52 [3.03; 4.01]	10 [7.85; 12.14]	13.77 [11.19; 16.34]	6.48	3.77	< 0.001
**Gender**
Male	3.87 [3.29; 4.44]	8.62 [6.49; 10.74]	13.32 [11.04; 15.61]	4.75	4.70	< 0.001
Female	5.01 [4.39; 5.62]	11.94 [9.85; 14.02]	17.88 [15.05; 20.71]	6.93	5.94	< 0.001
**Age groups (in years)**
25–34	1.84 [1.28; 2.40]	4.96 [2.55; 7.36]	9.04 [5.19; 12.88]	3.12	4.08	< 0.001
35–44	3.65 [2.89; 4.40]	8.8 [6.30; 11.30]	11.42 [8.53; 14.31]	5.15	2.62	< 0.001
45–54	6.6 [5.69; 7.51]	13.84 [10.77; 16.91]	19.58 [15.79; 23.37]	7.24	5.74	< 0.001
55–64	9.22 [8.06; 10.38]	17.72 [13.78; 21.66]	26.64 [23.15; 30.14]	8.50	8.92	< 0.001

There was no statistically significant difference in trends between rural and ruban (*p* = 0.72) and male and female (*p* = 0.78). However, the *p*-value for testing differences in trends among 4 age groups was statistical significant (*p* < 0.001). Among 4 age groups, the two oldest groups (aged 45–54 and 55–64 years) show the highest burden of elevated blood lipids in the year 2020 and the most significant changes over time. For the age group 45–54 years, the prevalence of elevated blood lipids went from 6.6% in 2010 to 19.58% in 2020 (a change of 12.98%). Among the group aged 55–64 years, this figure increased from 9.22% in 2010 to 26.64% (a change of 17.42%). and among four age groups (*p* = 0.44).

**Figure 4:**
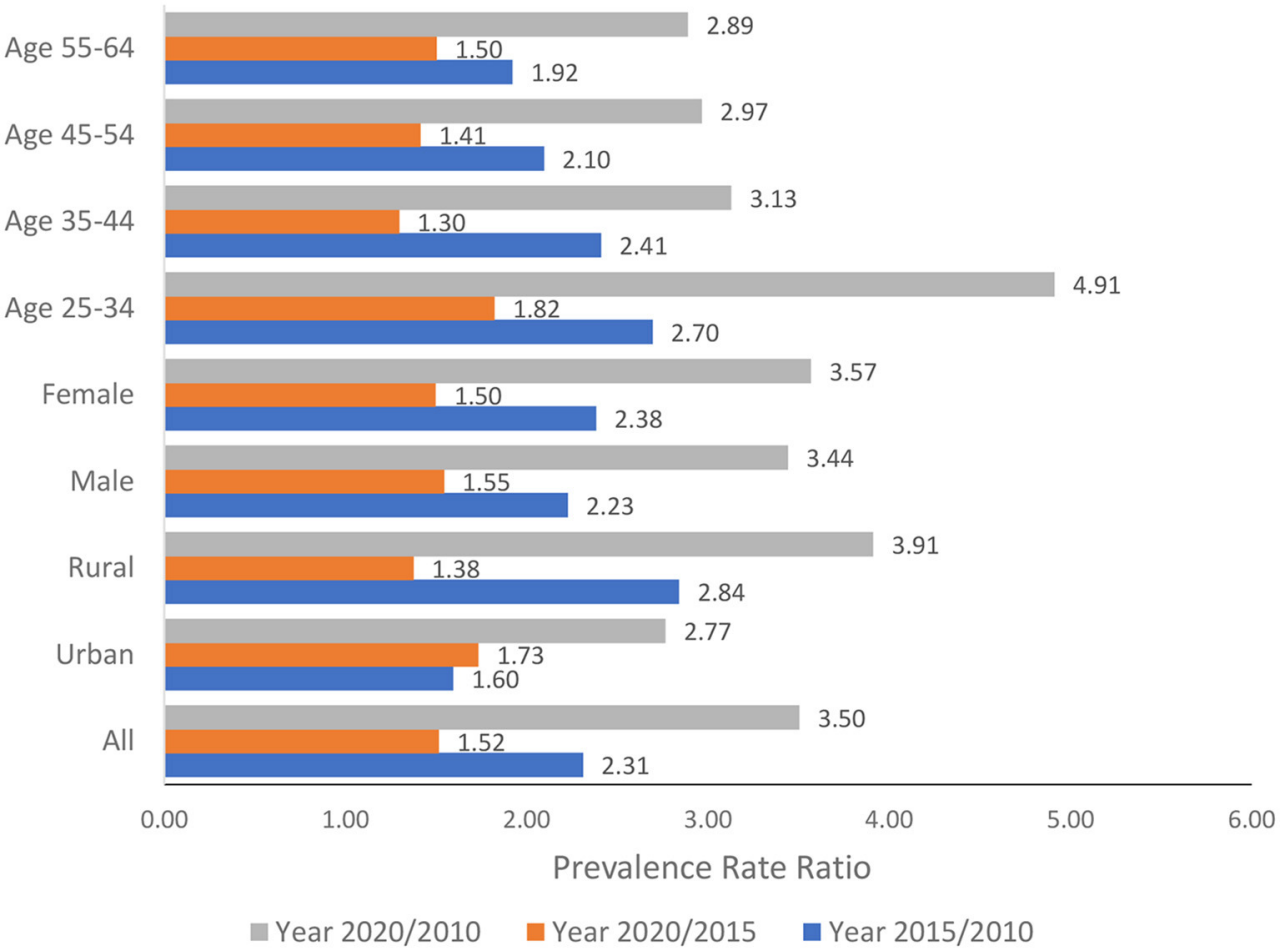
Relative changes in the prevalence of elevated blood lipids over time.

The PRRs of elevated blood lipids over time for all populations aged 25–64 years and all sub-group analyses were presented in Figure 4. The PRR for all populations between 2020 and 2010 for elevated blood lipids was 3.50, indicating that in 2020 the prevalence of elevated blood lipids increased 3.5 times compared to the year 2010. The relative changes over time were similar for the male and female populations. The rural population showed bigger relative changes in the burden of diseases over time compared to the urban population during the last 10 years (PRR 3.91 vs. 2.77).

When examining the absolute changes over time, two older groups (aged 45–54 years and 55–64 years) showed the sharpest increase. However, when examining the relative changes, two younger groups (aged 25–34 years and 35–44 years) demonstrated stronger changes in the prevalence ratio than the older groups. Most significant, the prevalence of elevated blood lipids in the year 2020 was 4.91 times higher than that in the year 2010 among the population aged 25–34 years.

**Figure 5:**
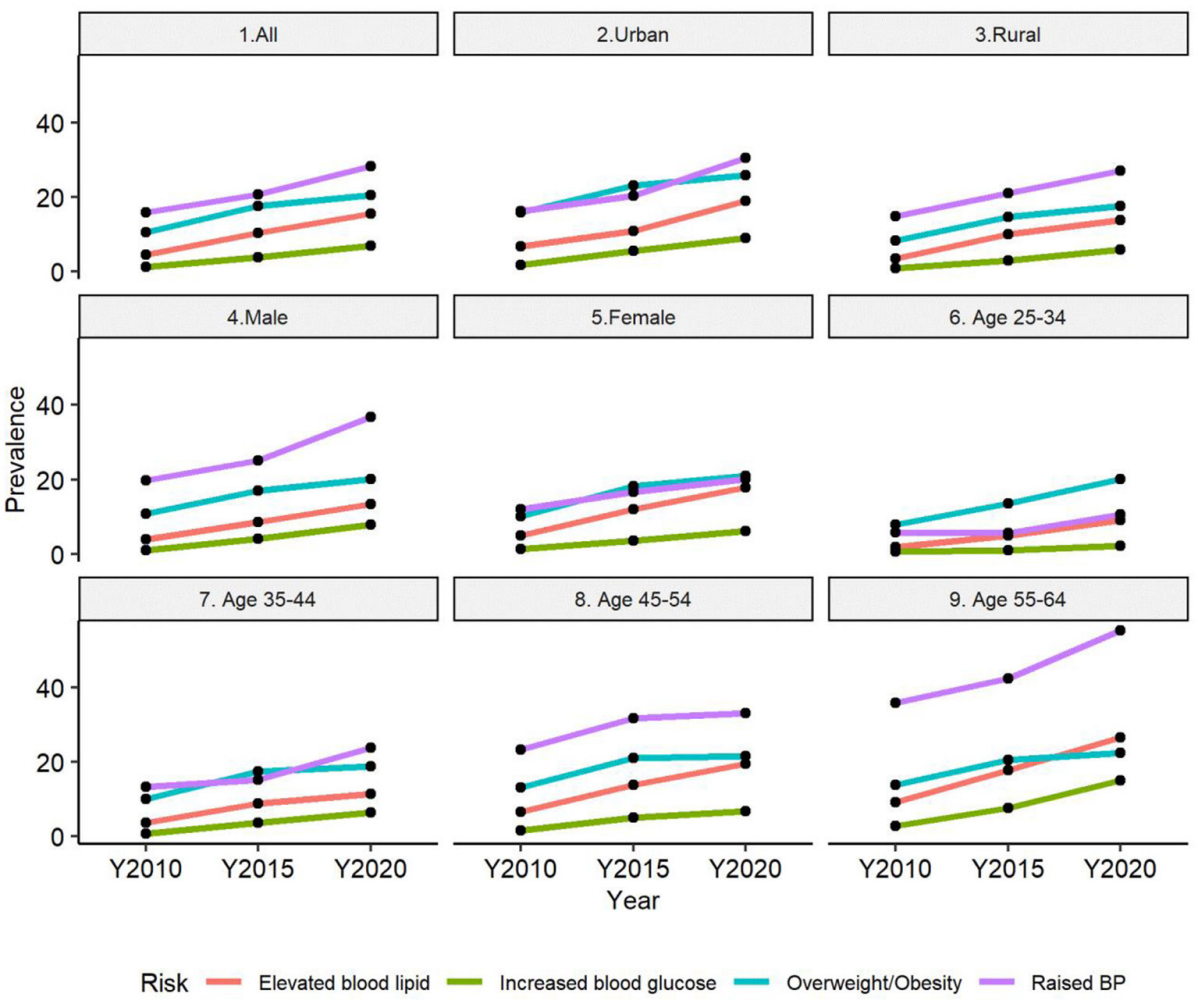
Trends of NCD metabolic risk factors over time across different population groups.

As visualized in Figure 5, the burden of all four risk factors has been increasing significantly over time among the studied population. For most groups (except the population aged 35–44 years and aged 45–54 years), the increasing burden of NCD metabolic risk factors was more significant during the period 2015–2020 compared to the period 2010–2015. The male population and population aged 55–64 years experienced the most dramatic changes in the burden of all NCD metabolic risk factors.

## Discussion

This study combined the data from the three national STEPs survey in Vietnam conducted during the last 10 years to examine the trend of 4 major NCDs metabolic risk factors for sampled population aged 25–64 years and explore these trends across different sub-population groups. Data provided point estimates and 95% CI for each NCDs risk factor at three-time points, a statistical test for the trend, and the relative changes in the burden of risk factors over time.

Globally, the age-standardized point prevalence of type 2 diabetes was highest in Oceania, Central Latin America, and the Caribbean (ranging from 7.5 to 11.9%) in 2019 (9). For raised BP, the prevalence was highest throughout central and Eastern Europe, central Asia, Oceania, southern Africa, and some countries in Latin America and the Caribbean, with the global average prevalence of 32% in women and 34% in men aged 30–79 years (10). In Vietnam, the prevalence of raised BP, overweight/obesity, increased BG, and elevated blood lipids among the population aged 25–69 years was 28.3, 20.57, 6.96, and 15.63%, respectively, in the year 2020. Compared to the global situation, Vietnam did not have the highest burden of raised BP and diabetes. However, it is important to note that the burden of NCDs among the older population (aged 55–64 years) was very high, as 55.26% lived with raised BP, 26.6% with elevated blood lipids, and 15.07% with diabetes.

Most recent estimations showed a stable or decreased trend in the global age-standardized prevalence of raised BP. For instance, a pooled analysis of 1,201 population-representative studies with 104 million participants for the period 1990–2019 estimated that the global age-standardized prevalence of raised BP in adults aged 30–79 years was stable during the last 20 years (around 32–34%) due to the net effect of a decrease in high-income countries and an increase in some low-income and middle-income countries. The highest increase was from 10 to 15 percentage points (absolute %) (10). Another study reported that the global age-standardized mean SBP in 2015 was stable among men and slightly declined among women ≥18 years of age since 1975. For both sexes, the age-standardized prevalence of raised BP declined globally, from 29.5 to 24.1% among men and from 26.1 to 20.1% among women during the period 1975–2015 (11). In Vietnam, the prevalence of raised BP among the population aged 25–64 years went from 15.85% in 2010 to 28.33% in 2020 (a change of nearly 13 percentage points). Thus, Vietnam currently does not have the highest burden of raised BP, but Vietnam is one of the countries with the most significant increasing trend of raised BP over time.

The prevalence of increased BG among the population aged 25–64 years in Vietnam increased from 1.19% in the year 2010 to 6.96% in the year 2020 (a net change of 5.85 percent point and a relative change of 4.9 times). This increasing trend was similar to other Asian countries. During the last 30 years, the incidence rate of increased BG in India increased by 110.9% and in China by 63.9% (12).

Previous studies attributed 80% of diabetes risk to an unhealthy diet, obesity, lack of exercise, and high triglyceride (13, 14). Multiple behaviors under modern lifestyles in Asian countries, such as exposure to industrial chemicals, smoking, and depression, were also indicated to have an impact on the high incidence of type 2 diabetes (15, 16).

The prevalence of elevated blood lipids increased sharply from 4.7% in 2010 to 15.6% in 2020. A recent global estimation pooled 1,127 population-based studies among 102.6 million people aged ≥18 and reported that the mean cholesterol levels did not change much over the four decades of follow-up. However, the trends in high-income and low-income countries are different, and high-income countries experience a significant decrease while low-income countries in Southeast Asia experience a most sharp increase in the mean of blood cholesterol (17).

The prevalence of overweight/obesity increased from 10.5% in 2010 to 20.6% in 2020. This trend was similar to other countries. For instance, the WHO European Region estimated that obesity prevalence rose by 21% in the 10 years before 2016 and by 138% since 1975; and for overweight (including obesity), by 8% in the 10 years before 2016 and by 51% since 1975 (18).

Globally, the increases in both plasma cholesterol and overweight/obesity were largely attributed to dietary habits and the adoption of unhealthy lifestyles (17). In Asia, these trends can be attributed to the increasing consumption of animal-source foods, refined carbohydrates, and palm oil (19, 20) and the low use of statin (21). Since 2010 Vietnam has seen a fast-food boom, with major chains like Pizza Hut, Domino's, Popeye's, Burger King, and KFC is entering the Vietnamese fast-food market (22). Many local brand names were also developed at the same time. This explanation was confirmed by the fact that the young population (aged 25–34 years), the main consumer of fast food in Vietnam, was the group that experienced the most significant relative changes in the prevalence of overweight/obesity (2.56 times higher) and elevated blood lipids (4.91 times higher).

Non-communicable diseases are affected by various factors, from nutritional and environmental to behavioral factors throughout the life course (11, 15, 23–25). To reverse the increasing trend of NCD metabolic factors in Vietnam, intervention and policy need to be applied to a comprehensive life course approach. For instance, intervention in early childhood, such as improved BMI and better nutrition, can shift the entire population's distribution of blood pressure and thereby change both the mean value and the prevalence of raised blood pressure (11).

This study was able to present the trends of 4 metabolic NCDs risk factors among population aged 25–64 years in Vietnam over time by combining data from three STEPs survey in 2010, 2015, and 2020. These trends provide important clues for the healthcare professionals to understand the unmet needs for care and the magnitudes of health problems (26). However, it should be noted that the three national surveys might have differences in sampling process, response rate and equipment for blood testing. Thus, all the interpretation of the results should consider these limitations.

## Conclusion

The study used three rounds of national STEPs surveys in Vietnam conducted in 2010, 2015, and 2020 to examine the trend of NCD metabolic risk factors for all populations aged 25–64 years and explore these trends across different sub-population groups. All NCD metabolic risk factors examined in this analysis showed significantly increasing trends over time. For most age groups, the increasing burden of NCD metabolic risk factors was more significant during the period 2015–2020 compared to the period 2010–2015. To reverse the increasing trend of NCD metabolic factors in Vietnam, intervention and policy need to apply a comprehensive life course approach.

## Data availability statement

The raw data supporting the conclusions of this article will be made available by the authors, without undue reservation.

## Ethics statement

The studies involving human participants were reviewed and approved by Ha Noi University of Public Health. The patients/participants provided their written informed consent to participate in this study.

## Author contributions

LV and QB designed and conceptualized the paper. LV and LK analyzed the data. All authors interpreted the results and prepared and reviewed the manuscript. All authors contributed to the critical revision of the manuscript for important intellectual content read and approved the final manuscript.

## Conflict of interest

The authors declare that the research was conducted in the absence of any commercial or financial relationships that could be construed as a potential conflict of interest.

## Publisher's note

All claims expressed in this article are solely those of the authors and do not necessarily represent those of their affiliated organizations, or those of the publisher, the editors and the reviewers. Any product that may be evaluated in this article, or claim that may be made by its manufacturer, is not guaranteed or endorsed by the publisher.
